# Obstructive Hypertrophic Cardiomyopathy and Interarterial Anomalous Aortic Origin of the Right Coronary Artery (AAORCA) Presenting With Syncope: Multimodal Diagnostic Assessment and Individualized Management

**DOI:** 10.7759/cureus.106361

**Published:** 2026-04-03

**Authors:** Jonathan Moyambi, Alfakihi Ahmed Said Ismail, Tagueniti Jalal, Brahim Meftout, Drighil Abdenasser

**Affiliations:** 1 Cardiology, Ibn Rochd University Hospital Center, Faculty of Medicine and Pharmacy, University Hassan II, Casablanca, MAR; 2 Cardiology, South Francilien Hospital Center, Corbeil-Essonnes, FRA

**Keywords:** anomalous aortic origin of the right coronary artery, implantable cardioverter-defibrillator, interarterial course, late gadolinium enhancement, mavacamten, multimodality imaging, obstructive hypertrophic cardiomyopathy, syncope, unexplained syncope

## Abstract

The coexistence of obstructive hypertrophic cardiomyopathy (oHCM) and anomalous aortic origin of the right coronary artery (AAORCA) with an interarterial course is exceptionally rare and poses a significant diagnostic and therapeutic challenge, particularly in patients presenting with syncope.

We report the case of a 63-year-old man referred for evaluation of a sudden syncopal episode that occurred at rest while he was seated on an airplane, followed by a fall on standing. Transthoracic echocardiography revealed asymmetric septal hypertrophy, systolic anterior motion of the mitral valve. and a marked dynamic obstruction of the left ventricular outflow tract (LVOT), with a peak gradient of 60 mmHg at rest and 123 mmHg during the Valsalva maneuver. Coronary CT angiography identified an anomalous origin of the right coronary artery from the left coronary sinus, with a proximal interarterial aortopulmonary course and a slit-like ostio-proximal segment, without intramural extension or significant stenosis. Cardiac MRI showed asymmetric septal hypertrophy with focal late gadolinium enhancement of the basal interventricular septum, consistent with myocardial fibrosis. The European Society of Cardiology (ESC) five-year sudden cardiac death risk score was 4.88%.

After multidisciplinary discussion, management included primary-prevention implantation of an implantable cardioverter-defibrillator, conservative management of the coronary anomaly, and initiation of mavacamten. Clinical and hemodynamic evolution was favorable, with no recurrence of syncope, no sustained ventricular arrhythmias on an implantable cardioverter-defibrillator (ICD) interrogation, and a marked reduction in the peak gradient of dynamic obstruction to 5 mmHg at rest and 7 mmHg after Valsalva at six-month follow-up.

This case illustrates the value of multimodal diagnostic evaluation and individualized management in patients with coexisting obstructive HCM and interarterial AAORCA. In this context, the integration of echocardiographic, computed tomography, magnetic resonance imaging, and clinical risk data can help guide both arrhythmic prevention and therapeutic decision-making.

## Introduction

Hypertrophic cardiomyopathy (HCM) and anomalous aortic origin of a coronary artery (AAOCA) are two uncommon conditions, each associated with a risk of syncope, myocardial ischemia, ventricular arrhythmias, and sudden cardiac death [1,2]. Their association is exceptional and may create significant diagnostic uncertainty, particularly when syncope may be related either to dynamic left ventricular outflow tract (LVOT) obstruction or to a potentially high-risk coronary anomaly [3-5].

Among the different forms of AAOCA, anomalous aortic origin of the right coronary artery (AAORCA) with an interarterial course is of particular clinical interest because its risk profile depends not only on the anomalous course itself but also on associated anatomical features, including an intramural segment, a slit-like ostium, and evidence of ischemia [2,6]. In parallel, obstructive hypertrophic cardiomyopathy (oHCM) may cause symptoms during exertion, or even at rest, because of marked dynamic LVOT obstruction, and is also associated with an increased arrhythmic risk, particularly in the presence of unexplained syncope, a family history of sudden cardiac death, or myocardial fibrosis [7].

We report the case of a 63-year-old man presenting with syncope at rest followed by a fall on standing, in whom multimodality imaging revealed the rare coexistence of oHCM and interarterial AAORCA. This case illustrates the difficulty of attributing symptoms, refining sudden cardiac death risk assessment beyond the calculated score alone, and tailoring management across three domains: arrhythmic prevention, treatment of LVOT obstruction, and conservative versus interventional management of the coronary anomaly.

## Case presentation

A 63-year-old man was referred to cardiology for the evaluation of a sudden syncopal episode that occurred at rest while he was seated on an airplane, without prodromal symptoms, followed by a fall on standing after a transient loss of consciousness. His family history was notable for the sudden death of his father at the age of 66 years, who had been followed for left ventricular hypertrophy suggestive of HCM.

**Figure 1:**
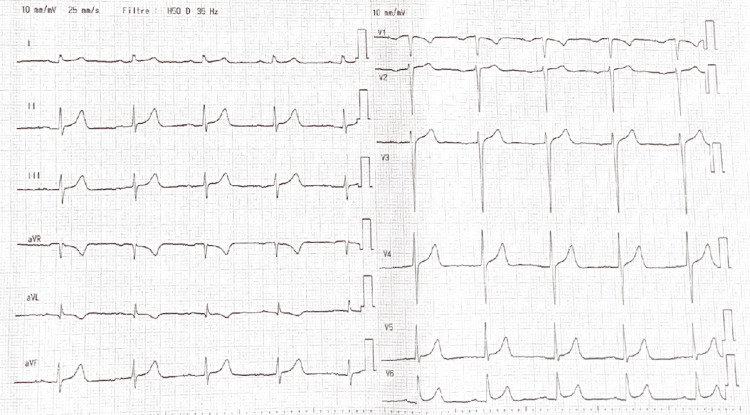
Baseline 12-Lead Electrocardiogram Twelve-lead electrocardiogram demonstrating normal sinus rhythm with no evidence of conduction abnormalities or acute repolarization changes.

His past medical history included hereditary hemochromatosis (C282Y mutation), hepatitis C treated in 2010, and recently diagnosed type 2 diabetes mellitus. At initial evaluation, the electrocardiogram (ECG) showed sinus rhythm without conduction or repolarization abnormalities (Figure 1).

**Figure 2:**
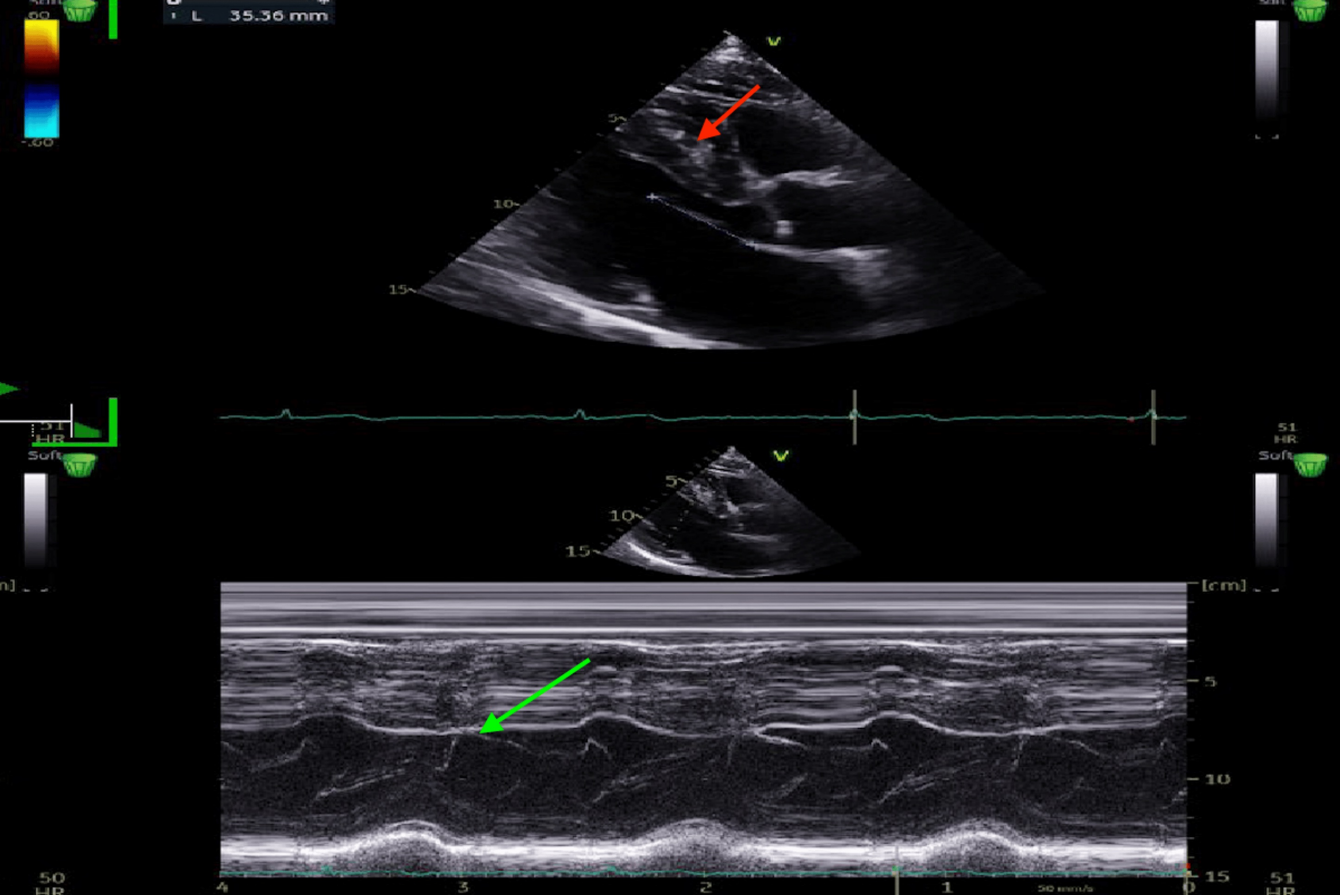
Parasternal Long-Axis M-Mode Transthoracic Echocardiography Parasternal long-axis M-mode transthoracic echocardiography demonstrating asymmetric septal hypertrophy (red arrow) and systolic anterior motion of the mitral valve (green arrow).

Transthoracic echocardiography revealed oHCM with asymmetric septal hypertrophy, a maximal septal thickness of 18 mm, systolic anterior motion of the mitral valve, and dynamic LVOT obstruction (Figure 2).

**Figure 3:**
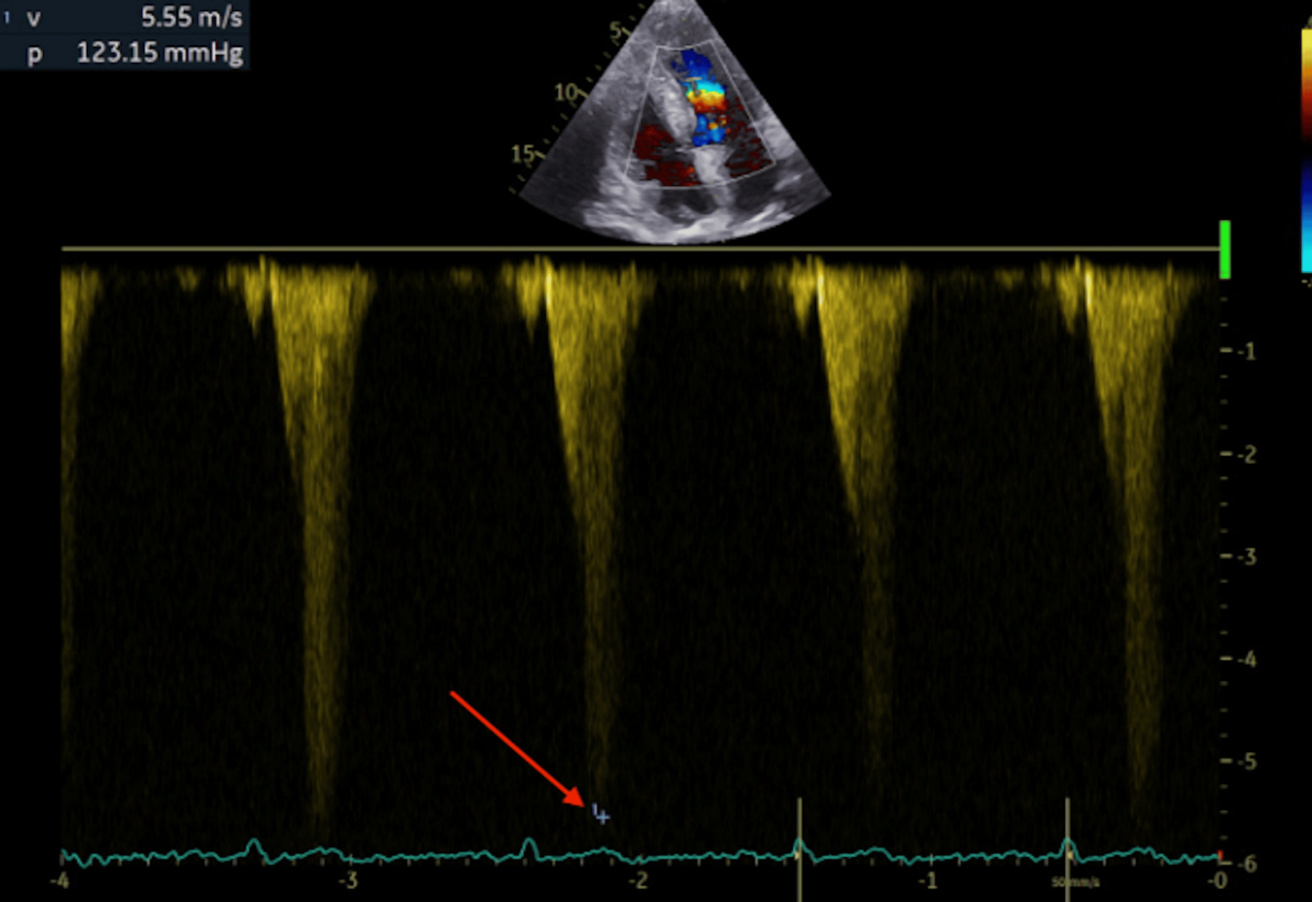
Continuous-Wave Doppler Assessment of Dynamic LVOT Obstruction Continuous-wave Doppler aligned with the aliasing jet demonstrates a late-peaking dagger-shaped systolic envelope consistent with dynamic LVOT obstruction, with a peak velocity of 5.55 m/s and an estimated peak gradient of 123 mmHg (red arrow)

The maximum gradient was 60 mmHg at rest and increased to 123 mmHg during the Valsalva maneuver on successive assessments with preserved left ventricular systolic function (Figure 3).

Twenty-four-hour Holter monitoring showed sinus rhythm, with a mean heart rate of approximately 54 bpm, frequent premature atrial contractions (approximately 1,030/24 h), and a moderate burden of premature ventricular contractions (approximately 1,179/24 h). No sustained atrial fibrillation, R-on-T phenomenon, or clinically significant conduction abnormalities were observed.

**Figure 4:**
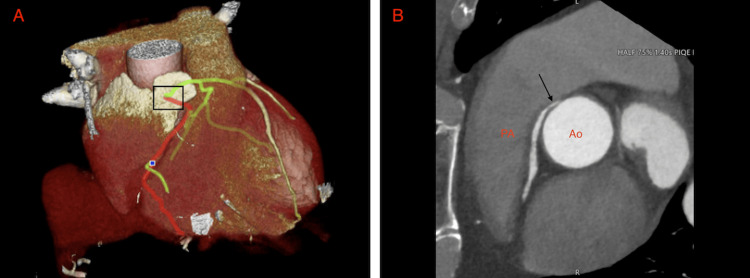
Coronary CT Angiography Showing Interarterial AAORCA Coronary CT angiography demonstrating anomalous origin of the right coronary artery from the left coronary sinus, with a proximal interarterial course between Ao and PA. (A) Three-dimensional volume-rendered image demonstrating anomalous origin of the right coronary artery (red) from the left coronary sinus, arising on the same side as the left main coronary artery(green). (B) Axial image showing the anomalous origin of the right coronary artery (black arrow) from the left coronary sinus. The proximal right coronary segment is markedly small in caliber, without intramural extension, and resumes a normal distal caliber, with no significant stenosis. AAORCA: Anomalous aortic origin of the right coronary artery; Ao: aorta; PA: pulmonary artery.

Coronary CT angiography confirmed oHCM and identified an associated congenital coronary anomaly: anomalous origin of the right coronary artery from the left coronary sinus, with a proximal interarterial aortopulmonary course and a slit-like ostio-proximal segment, without an intramural course or significant stenosis. The left coronary system was dominant and free of significant stenosis (Figure 4).

**Figure 5:**
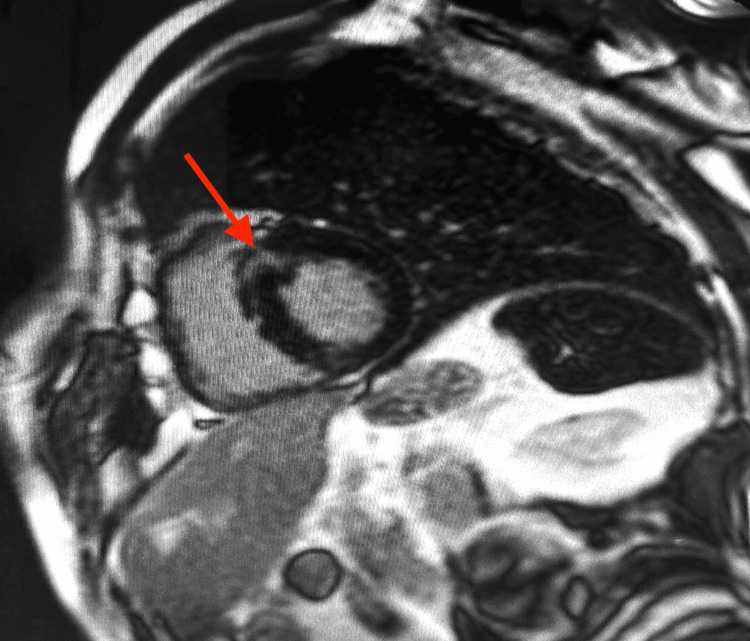
Cardiac Magnetic Resonance Late Gadolinium Enhancement of the Basal Septum PSIR late gadolinium enhancement cardiac magnetic resonance image in the basal short-axis view demonstrating focal hyperenhancement in the basal septum (red arrow), consistent with myocardial fibrosis.

Cardiac magnetic resonance imaging showed asymmetric HCM with predominant septal involvement, along with focal intramyocardial late gadolinium enhancement of the basal interventricular septum, consistent with myocardial fibrosis (Figure 5). The European Society of Cardiology (ESC) five-year sudden cardiac death risk score was calculated at 4.88%.

After multidisciplinary discussion, management included primary-prevention implantation of an implantable cardioverter-defibrillator (ICD), conservative management of AAORCA in the absence of an intramural segment, anginal symptoms, or convincing evidence of ischemia, and medical treatment of obstructive HCM with mavacamten 5 mg/day, bisoprolol 2.5 mg/day (dose reduced because of bradycardia-related intolerance), dapagliflozin, and metformin. Before initiation of mavacamten, CYP2C19 pharmacogenetic testing was performed and showed a normal metabolizer phenotype, allowing treatment to be started according to the standard algorithm with close echocardiographic monitoring.

**Figure 6:**
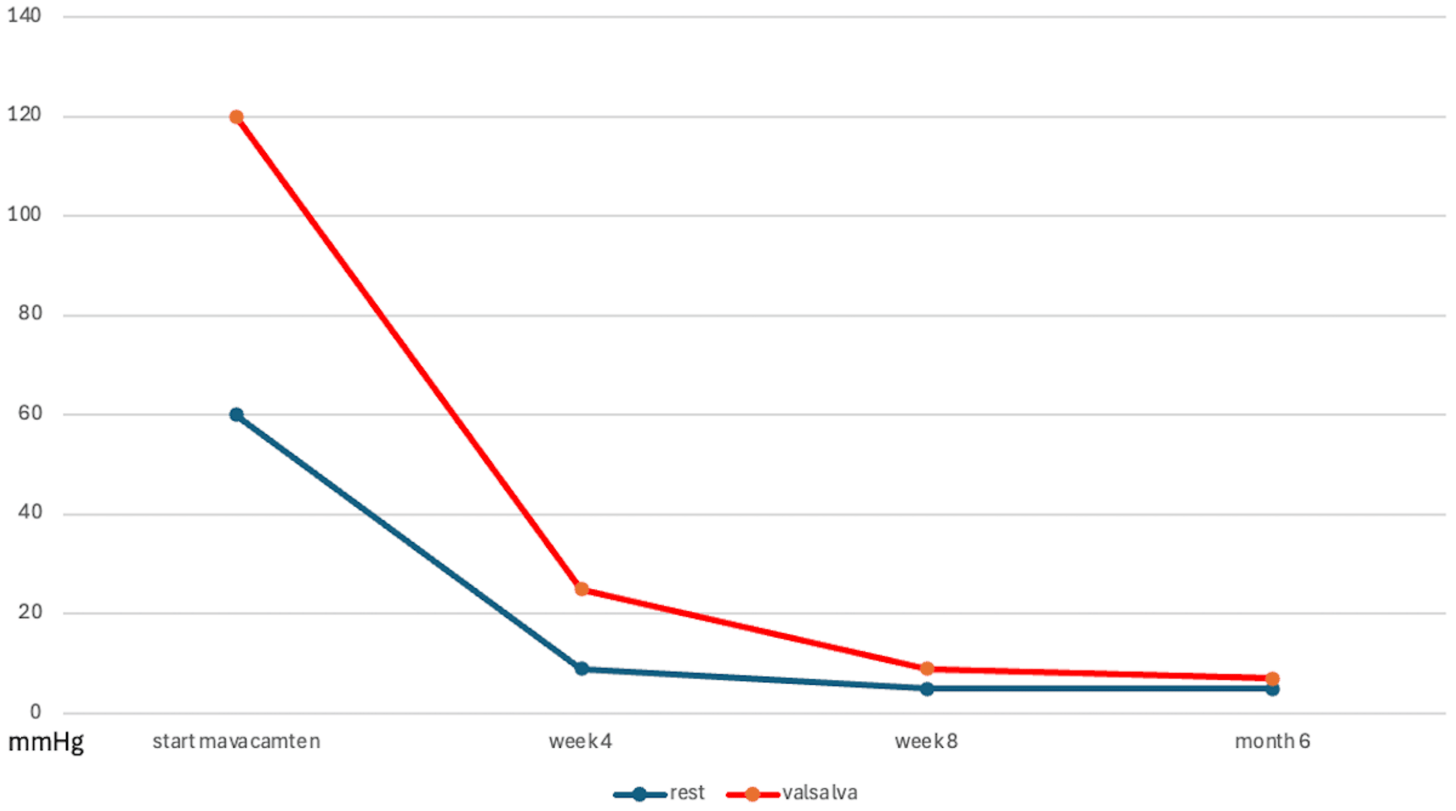
Evolution of the Peak Gradient of Dynamic LVOT Obstruction After Mavacamten Initiation Evolution of the peak gradient of dynamic LVOT obstruction measured by transthoracic echocardiography at rest (blue line) and during the Valsalva maneuver (red line), showing a marked and sustained reduction after initiation of mavacamten. LVOT: Left ventricular outflow tract. Image credit: Created by Dr. Jonathan Moyambi using Microsoft Excel (Microsoft, Redmond, WA).

**Figure 7:**
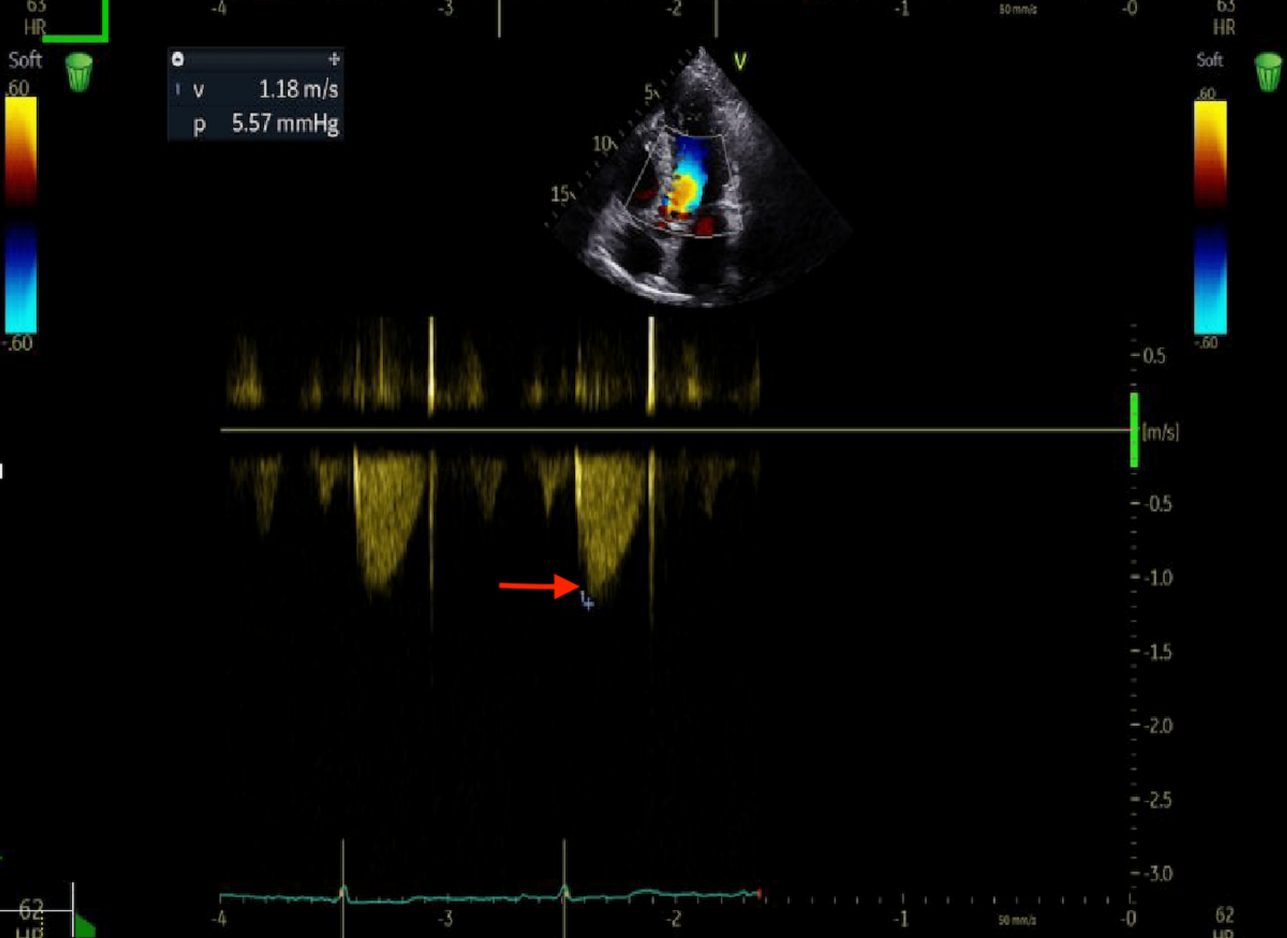
Follow-Up Continuous-Wave Doppler at Six Months Follow-up transthoracic echocardiography with continuous-wave Doppler demonstrating minimal residual LVOT dynamic obstruction at rest. The red arrow marks the peak of the systolic envelope, corresponding to a peak gradient of 5 mmHg at six-month follow-up. LVOT: Left ventricular outflow tract.

Clinical and hemodynamic evolution was favorable. Serial transthoracic echocardiography showed a marked and sustained reduction in the peak gradient of dynamic obstruction over follow-up (Figure 6). At week 4 after initiation of mavacamten, peak gradients were 9 mmHg at rest and 25 mmHg after the Valsalva maneuver, while left ventricular ejection fraction remained preserved. At week 8, peak gradients were 5 mmHg at rest and 9 mmHg after Valsalva, with preserved left ventricular systolic function. At six-month follow-up, the patient was in the New York Heart Association (NYHA) functional class I, with no recurrence of syncope or signs of heart failure; echocardiography showed peak gradients of 5 mmHg at rest and 7 mmHg after Valsalva, with preserved left ventricular systolic function (Figure 7).

**Figure 8:**
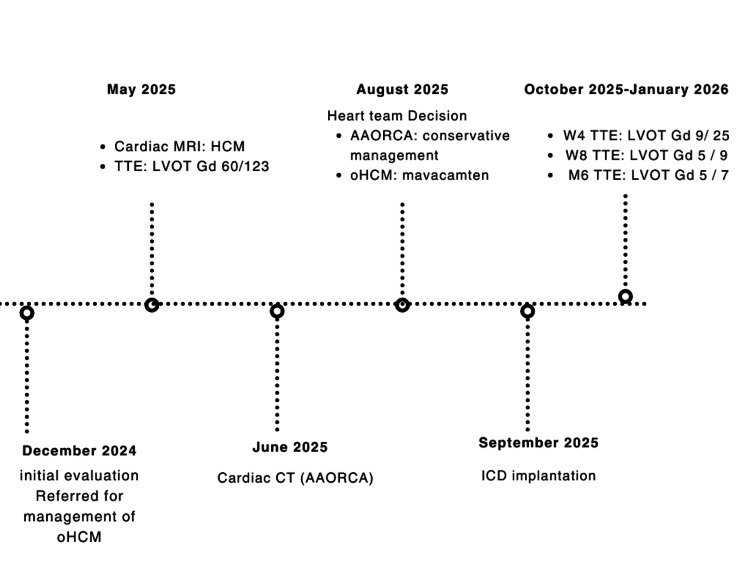
Timeline of the Main Clinical, Diagnostic, and Therapeutic Events Chronological timeline summarizing the main clinical, diagnostic, and therapeutic events from December 2024 to January 2026. Abbreviations: ECG, electrocardiogram; TTE, transthoracic echocardiography; CT, computed tomography; CMR, cardiac magnetic resonance; LGE, late gadolinium enhancement; LVOT, left ventricular outflow tract; Gd, gradient; oHCM, obstructive hypertrophic cardiomyopathy; HCM, hypertrophic cardiomyopathy; AAORCA, anomalous aortic origin of the right coronary artery; ICD, implantable cardioverter-defibrillator; W, week; M, month. Image credit: Created by Dr. Jonathan Moyambi using the free version of Canva (Canva, Sydney, Australia).

No sustained ventricular arrhythmias were detected during ICD interrogation. The overall clinical course is summarized in the timeline (Figure 8).

## Discussion

The coexistence of oHCM and AAORCA with an interarterial course is rarely reported and remains a major diagnostic challenge in cases of syncope [3,5]. In this setting, the key challenge is to determine the most likely mechanism underlying the clinical presentation and, consequently, to guide therapeutic priorities.

In our patient, several findings supported a predominant role of obstructive HCM in the onset of syncope. Echocardiography showed asymmetric septal hypertrophy, systolic anterior motion of the mitral valve, and, most importantly, marked dynamic LVOT obstruction, with a gradient of 60 mmHg at rest and 123 mmHg with Valsalva. Dynamic LVOT obstruction is a major determinant of symptoms in obstructive HCM and can contribute to presyncope or syncope by abruptly reducing forward stroke volume under changing loading conditions [8,9]. Furthermore, the significant reduction in gradients at six months after initiation of mavacamten, together with the absence of recurrent syncope during follow-up, further supports the hypothesis that obstruction played a central role in the initial presentation [10].

However, the coronary anomaly could not be dismissed initially. Interarterial AAORCA is generally considered a potentially high-risk anatomical variant, particularly when associated with unfavorable features such as an intramural segment, a slit-like ostium, or documented ischemia [2,6]. In our case, coronary CT angiography provided precise anatomical characterization, revealing a proximal interarterial course with a slit-like ostio-proximal segment, but without an intramural segment or hemodynamically significant stenosis. There was no angina and no convincing clinical or imaging evidence to suggest ischemia as the main mechanism of presentation. In this context, conservative management of the coronary anomaly was considered appropriate after multidisciplinary discussion, with long-term follow-up.

The ESC five-year sudden cardiac death risk score in HCM was 4.88%, corresponding to an intermediate-risk profile. However, risk assessment in HCM should not rely solely on the numerical value of the score when additional clinical or morphological modifiers are present [7,11]. In our patient, unexplained syncope, family history of sudden death, and the presence of septal late gadolinium enhancement on cardiac MRI all supported a more cautious approach. Although the extent of late gadolinium enhancement was not quantified as a percentage of left ventricular mass, its presence contributed to the overall arrhythmic risk profile and supported the decision for primary-prevention ICD implantation after multidisciplinary discussion [7,12].

Multimodality imaging was central to this strategy. Echocardiography allowed identification of the obstructive phenotype and quantification of the hemodynamic burden of obstruction. Coronary CT angiography precisely defined the origin and course of the anomalous right coronary artery, while ruling out an intramural segment. Cardiac MRI confirmed the hypertrophic phenotype and demonstrated focal septal fibrosis [5,12]. Integration of these modalities allowed a more accurate interpretation of syncope and more individualized management.

From a therapeutic perspective, the use of beta-blockers was limited by bradycardia-related intolerance, and persistence of significant obstruction led to initiation of mavacamten. This selective cardiac myosin inhibitor has been shown to reduce gradients and improve symptoms in symptomatic obstructive HCM [10]. In our patient, the marked reduction in the obstructive gradient and the parallel clinical improvement support the role of dynamic obstruction as the predominant mechanism and the value of targeted therapy in this complex phenotype.

This case suggests that when obstructive HCM coexists with interarterial AAORCA, management should be guided by integrated clinical reasoning tailored to the individual patient. In our patient, the absence of intramural anatomy and convincing evidence of ischemia justified a conservative approach to the coronary anomaly, whereas the combination of unexplained syncope, family history, myocardial fibrosis, and obstructive physiology supported primary-prevention ICD implantation and targeted anti-obstructive therapy. Long-term follow-up remains essential, given the potentially persistent and incompletely characterized risk associated with the coexistence of these two rare conditions.

## Conclusions

This case illustrates the diagnostic complexity of syncope in a patient with coexisting oHCM and interarterial AAORCA. Both conditions can theoretically contribute to symptoms and sudden cardiac death risk, and management required an integrated assessment combining echocardiography, coronary CT angiography, cardiac MRI, rhythm evaluation, and clinical risk markers. In our patient, the absence of intramural anatomy, significant stenosis, or convincing evidence of ischemia supported a conservative approach to the coronary anomaly. However, the severity of dynamic LVOT obstruction, unexplained syncope, family history of sudden death, and the presence of septal late gadolinium enhancement supported initiation of mavacamten and primary-prevention ICD implantation after multidisciplinary discussion.

The subsequent clinical and hemodynamic evolution further supported this interpretation. The marked reduction in LVOT gradient after initiation of mavacamten, together with the absence of recurrent syncope, suggests that obstruction played a predominant role in the initial presentation. Overall, this case underscores the value of individualized multimodal assessment, tailored management, and long-term follow-up in this rare phenotype.
